# Lichens as Biomonitors of Air Quality and Climate

**DOI:** 10.1111/gcb.70768

**Published:** 2026-02-23

**Authors:** Claudia Colesie, Kevin K. Newsham

**Affiliations:** ^1^ The University of Edinburgh, School of Geosciences, Global Change Research Institute Edinburgh UK; ^2^ British Antarctic Survey, Natural Environment Research Council Cambridge UK

**Keywords:** air pollution, air temperature, ammonia (NH_3_), epiphytic lichens, nitrogen oxides (NO_x_), relative humidity, sulphur dioxide (SO_2_)

Lichens—morphologically and physiologically integrated symbioses between at least one fungus and at least one phototroph (typically a green alga or a cyanobacterium)—are among the most iconic and widespread symbiotic organisms, and are widely used as indicators of environmental quality. Since the late 19th century, surveys of epiphytic lichen communities (i.e., those growing on tree bark) have been used to estimate air pollution levels. These surveys are based on differing sensitivities of lichen species to atmospheric pollutants, and particularly sulphur and nitrogen compounds (Davies et al. 2007; Greaver et al. 2023; Hawksworth and Rose 1970). Recently, lichens have also been found to be highly responsive to rising air temperatures associated with global warming, offering promise to detect biological impacts of climate change in the natural environment on these slow‐growing, long‐lived organisms (Aptroot and van Herk 2007; Sancho et al. 2019; Stapper and John 2015).

Just over a decade ago, a European standard (EN 16413:2014 Ambient air—Biomonitoring with lichens—Assessing epiphytic lichen diversity) was adopted by the Comité Européen de Normalization to establish reliable, consistent and objective methods to assess epiphytic lichen diversity (Cristofolini et al. 2014). By specifying aspects of plot allocation, tree selection and methods for assessing lichen diversity, the standard aimed to enhance data quality and comparability across studies. It used lichen species richness as a measure of environmental quality, with higher richness values indicating lower air pollution or habitat disturbance. However, field evaluations of the standard have revealed substantial sources of error, even among experienced lichenologists. These errors arise primarily from difficulty in locating a plot's centre, tree selection and accurate lichen species identification (Cristofolini et al. 2014). In addition, the standard cannot disentangle lichen responses to individual pollutants, limiting its broader applicability and hindering the widespread adoption of a standardized protocol.

Against this backdrop, in this edition of *Global Change Biology* Counoy et al. (2025) initiate the development of a refined standardized framework for European lichen biomonitoring. They examine data from 58 studies using the European standard on 9064 trees at 2932 sites in 15 countries. From an initial pool of 477 lichen species, the authors identify a core subset of 43 species exhibiting consistent responses to sulphur dioxide (SO_2_), ammonia (NH_3_) and nitrogen oxides (NO_x_), and mean air temperature, relative humidity and temperature seasonality. For each of the 43 species, the authors helpfully include information on whether it can be easily recognised in the field and distinguished from similar taxa. In order to reduce identification bias, morphologically similar species are aggregated together into the same taxonomic units. For example, *Physcia tenella* (Figure 1a) and *P. adscendens* (Figure 1b) are grouped into *Physcia* gr. *adscendens*. Since bark pH strongly affects lichen community composition (Hawksworth and Rose 1970), the study is restricted to tree species with acid to subneutral bark, and the analyses are further restricted to data from open areas such as parks, where lichens are more likely to be directly affected by air pollution. Quantile regressions, which generate median prediction fits that do not always intuitively match the observed data pattern, are used to estimate the environmental conditions under which each lichen species reaches its highest frequency.

**FIGURE 1:**
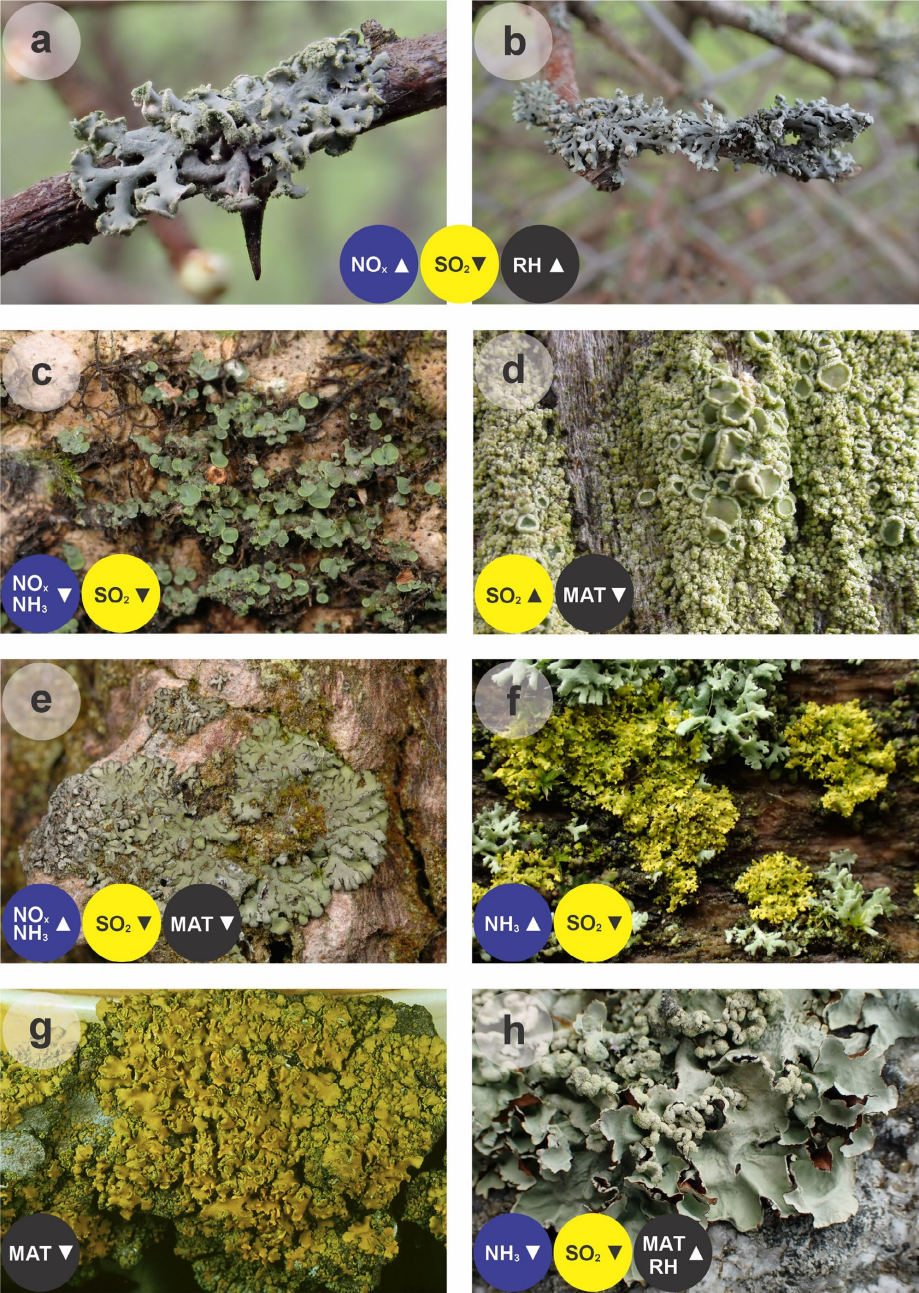
Eight of the 43 lichen indicator species identified by Counoy et al. (2025) and their responses to air pollutants and climate variables. (a) *Physcia tenella* , (b) *P. adscendens* , (c) *Normandina pulchella*, (d) *Lecanora conizaeoides* , (e) *Phaeophyscia orbicularis* , (f) *Candelaria concolor* (yellow), (g) *Xanthomendoza fallax* and (h) *Parmotrema perlatum*. Note that Counoy et al. (2025) group *Physcia tenella* and *P. adscendens* into *Physcia* gr. *adscendens* to reduce identification bias. Blue, yellow and black circles show the responses of each species to NO_x_ or NH_3_, SO_2_ and climate variables, respectively, with upwards‐ and downwards‐pointing arrowheads indicating increases and decreases in the frequency of each species, respectively. RH, relative humidity; MAT, mean air temperature. Images in panels (a), (b), (f) and (h) copyright Rebecca Yahr, images in (c) and (e) copyright Mike Sutcliffe, image in (d) copyright Mark Powell and image in (g) copyright Ulrik Søchting.

Congruent with previous findings, Counoy et al. (2025) report that SO_2_—the main gas forming acid rain, which is predominantly generated by the combustion of coal and oil—has the most consistent negative effect on the frequencies of lichens. Where SO_2_ is still prevalent, such as in the cities of Antwerp and Marseille, 28 of the 43 indicator species show negative responses to the gas. These include *Normandina pulchella* (Figure 1c), *Evernia prunastri* , *Parmelina tiliacea*, and *Physconia grisea* , species well known to be sensitive to SO_2_ (Hawksworth and Rose 1970). In contrast, the frequencies of only four species— *Lecanora conizaeoides* (Figure 1d), *L*. gr. *expallens*, *Amandinea punctata*, and *Hypogymnia tubulosa* —show positive associations with atmospheric SO_2_ concentrations. Notably, the former three species were among those able to survive exposure to SO_2_ concentrations of > 125 μg m^−3^ in the so‐called ‘lichen deserts’ of city centres in the mid–late 20th century (Hawksworth and Rose 1970).

Counoy et al. (2025) also report the effects of oxidized and reduced forms of atmospheric nitrogen pollutants on lichens. Typically, increasing concentrations of NO_x_—chiefly nitric oxide (NO) and nitrogen dioxide (NO_2_), which arise from the combustion of fossil fuels—negatively affect lichens. Of the 43 indicator species, 15 decline in frequency at high NO_x_ concentrations, with only *Phaeophyscia orbicularis* (Figure 1e), *Physconia grisea* and *P*. gr. *adscendens* increasing in frequency as NO_x_ concentrations rise in the atmosphere. In contrast, NH_3_, which is volatilized from livestock urine and manure, has more frequent positive effects on the occurrence of lichens, with eight species responding positively to the gas, including the classic nitrophiles *Candelaria concolor* (Figure 1f), *P. orbicularis* and *Physconia grisea* . The latter two species respond positively to both NO_x_ and NH_3_, whilst 10 others, including *N. pulchella* , respond negatively to both gases. However, Counoy et al. (2025) find *Arthonia radiata* and *Xanthoria parietina* to respond negatively and positively to NO_x_ and NH_3_, respectively. The reasons for the contrasting effects of the pollutants on these two lichen species remain obscure, but serve to underline the complexities of disentangling the impacts of oxidized and reduced forms of nitrogen on lichens in the natural environment (Greaver et al. 2023).

Because lichens grow continuously over long periods—with some Antarctic species, such as *Buellia frigida*, surviving for nearly 6000 years (Sancho et al. 2019)—they integrate environmental conditions over extended timescales. This makes them particularly valuable indicators of gradual climate change (Sancho et al. 2019). Here, Counoy et al. (2025) show species such as *Xanthomendoza fallax* (Figure 1g), *Polycauliona candelaria*, and *P. polycarpa* to be more frequent in cooler climates, whereas species including *Parmotrema perlatum* (Figure 1h), *Hyperphyscia adglutinata* , and *Ramalina farinacea* are shown to be associated with warmer conditions and higher relative humidity. In support of these findings, the frequencies of the latter three species each increased on host trees in Düsseldorf between 2001 and 2013 (Stapper and John 2015), raising the prospect that they could be used as biomonitors of climate change (Aptroot and van Herk 2007).

Data interpretation in many environmental studies is hindered by multicollinearity between explanatory variables (Dormann et al. 2013). In an attempt to circumvent this problem, Counoy et al. (2025) exclude particulate matter and ozone as explanatory variables from their analyses because they are closely correlated with other variables. Even so, their models indicate that for all but three species (*Lecanora allophana, Lepraria* sp. and *X. fallax* ) lichen frequency was influenced by more than one variable. In the complex dataset compiled by the authors, disentangling the effects of variables on the remaining 40 species—and, importantly, establishing the statistical significance of each variable—pose significant challenges. The application of methods such as penalized regressions, which can deal with multicollinearity and are used for variable selection (Dormann et al. 2013), may help to identify the best explanatory variable for each species. However, the presence of numerous zeroes in the dataset, caused by the absence of lichens from surveyed trees, is likely to complicate the application of these methods. Ultimately, the most certain way of determining how individual variables affect the indicator species would be to conduct field experiments in which the frequency of each species exposed to controlled levels of SO_2_, NH_3_ or NO_x_, or altered temperature and humidity, is recorded over decadal time scales (e.g., Neufeld and Perkins 2021).

In summary, Counoy et al. (2025) make an important methodological advance in lichen biomonitoring. Whilst the proposed framework is not without its limitations, it represents a solid foundation on which future standardized lichen monitoring in Europe, and perhaps other continents, can be built. In order to improve consistency and comparability among studies, the authors advocate that *Acer*, *Fraxinus*, *Quercus*, and *Tilia* species are prioritized as host trees in future monitoring, that morphologically similar lichen species (such as *Physcia tenella* and *P. adscendens* ) are grouped together, and that pioneer or inconspicuous lichen species are excluded from future surveys. By implementing these changes, they conclude that future monitoring programs and comparisons between studies will be improved, leading to the development of actionable air quality indices and the refinement of region‐scale analyses, supporting a harmonized framework of lichen biomonitoring across Europe.

## Author Contributions

**Claudia Colesie:** writing – review and editing. **Kevin K. Newsham:** writing – review and editing.

## Conflicts of Interest

The authors declare no conflicts of interest.

## Linked Articles

This article is an invited commentary on Counoy et al., https://doi.org/10.1111/gcb.70632.

## Data Availability

Data sharing not applicable to this article as no datasets were generated or analysed during the current study.
